# MiR-320a contributes to atherogenesis by augmenting multiple risk factors and down-regulating SRF

**DOI:** 10.1111/jcmm.12483

**Published:** 2015-02-27

**Authors:** Chen Chen, Yan Wang, Shenglan Yang, Huaping Li, Gang Zhao, Feng Wang, Lei Yang, Dao Wen Wang

**Affiliations:** a Department of Internal Medicine and Gene Therapy Center, Tongji Hospital, Tongji Medical College, Huazhong University of Science and TechnologyWuhan, China; b Department of Internal Medicine, The First Affiliated Hospital of Chongqing Medical UniversityChongqing, China

**Keywords:** atherosclerosis, miRNA, SRF, endothelium

## Abstract

Atherosclerosis progress is regulated by a variety of factors. Here, we show that miR-320a, an intergenic miRNA, is markedly elevated in the peripheral blood of coronary heart disease patients and high-risk patients. Microarray analysis and qRT-PCR assays showed that circulating miRNA-320a was highly expressed in coronary artery disease patients. *In vivo* study showed that overexpression of miR-320a resulted in significant increase in levels of plasma lipid (total cholesterol, Triglyceride and low-density lipoprotein) and serum inflammatory cytokines (IL-6, MCP-1, sICAM, pSelectin, TNF-α and fibrinogen). In ApoE^−/−^ mice, miR-320a expression attenuates endothelium cell function and promotes atherogenesis. Bioinformatics analysis identified serum response factor as a potential target for miR-320a, which was validated by luciferase reporter activity assay and western-blot *in vitro* and *in vivo*. Moreover, miR-320a expression inhibits human-derived endothelium cell proliferation and induces apoptosis. We also found that SP1 transcriptionally up-regulates hsa-miR-320a expression. Our observations indicate that miR-320a is a key regulator contributing to multiple aspects of atherogenesis.

## Introduction

Atherosclerosis-associated vascular diseases are the most prevalent cause of death worldwide. The genesis and progression of atherosclerosis are complex and multi-factorial. Endothelial injury appears to play important role in the initial step of atherogenesis. Plasma lipids and pro-inflammatory cytokines are also well-recognized risk factors 1.

MicroRNAs (miRNAs) are small endogenous 21–23 nucleotide double-stranded RNAs playing roles in diverse physiological and pathological processes 2. MiRNAs negatively regulate gene expression through two major mechanisms: translational repression and mRNA cleavage/degradation, by the ability to bind to its mRNA targets and other criteria still being defined 3. Recently several miRNAs have been implicated in cardiac pathology such as cardiac hypertrophy 4, arrhythmia 5, fibrosis 6, ischemia/reperfusion 7 and vascular diseases such as post-angioplasty restenosis 8 and angiogenesis 9. MiRNAs were identified playing important roles in atherosclerotic cardiovascular diseases 10–13. However, the underlying mechanisms were still unclear.

Serum response factor (SRF) is a MADS (The MADS-box gene family got its name later as an acronym referring to the four founding members: MCM1 from the budding yeast, AGAMOUS from the thale cress, DEFICIENS from the snapdragon, SRF from the human. It dose not have a full name.) box transcription factor critical in regulating cell growth and differentiation 14. As a key cardiac transcription factor, SRF is involved in muscle contraction 15, actin cytoskeleton 16, myosin 17, and sarcomere function 18. Transcriptional activation of muscle-specific genes requires cooperation between SRF and its co-activators. In addition, SRF is a key regulator for endothelial cell function and is essential for VGEF signalling. SRF itself is regulated by cell signalling pathways and interaction with other transcription factors 19. The process is complex and likely involving post-transcriptional mechanisms, *e.g*., recent report showed that SRF may regulate miRNA expressions. At least 169 miRNAs in mammalian genomes contain at least one SRF binding element CArG in their promoter region 20. On the other hand, some miRNAs can also regulate SRF synthesis 21, therefore forming intricate and complex regulatory web.

Here, we provide evidence that miR-320a is a key regulator of atherogenesis. MiR-320a overexpression promotes atherogenesis by augmenting multiple risk factors of coronary artery disease (CAD).

## Materials and methods

### Ethics statement

The institutional review board of Tongji hospital approved this study. Written informed consents were obtained from all participants. Experiments were conducted according to the principles expressed in the Declaration of Helsinki and the NIH Belmont Report.

All mouse studies were conducted with the approval of the Animal Research Committee of Tongji Medical College, and in accordance with the NIH *Guide for the Care and Use of Laboratory Animals*.

### Materials

Chemicals were obtained from Sigma-Aldrich Shanghai, China unless otherwise noted. Cell culture medium and foetal bovine serum (FBS) were obtained from Invitrogen (Life Technologies Corporation, Carlsbad, CA, USA). Mouse monoclonal antibodies against MoMa-2 and α-actin were purchased from Abcam Inc. (Cambridge, MA, USA). Mouse monoclonal antibodies against SRF, Sp1, ERK2, JNK and β-actin were purchased from Santa Cruz Biotechnology, Inc. (Santa Cruz, CA, USA). Horseradish peroxidase-conjugated secondary antibody (goat antimouse IgG) and enhanced chemiluminescence reagents were purchased from Pierce, Inc. (Thermo Fisher Scientific Inc., Rockford, IL, USA). DNA ladders, prestained protein markers and protein extraction kit were purchased from Fermentas Inc. (Thermo Fisher Scientific Inc., Glen Buenie, MD, USA). Polyvinylidene difluoride (PVDF) membranes were obtained from Millipore (Merck KGaA, Darmstadt, Germany). miRNA mimics (synthesized double-stranded), miRNA random control (little homology to any human, mouse or rat miRNAs annotated in the current released of the miRBase database, and no significant homology to the genomes of those three species as established by sequence alignment), miRNA inhibitors (antisense oligonucleotides) and siRNAs were commercial double-stranded oligonucleotides products chemically synthesized by Ribobio Co. (Guangzhou, China). All other reagents were purchased from standard commercial suppliers unless otherwise indicated.

### Recruitment for the discovery samples

Coronary artery disease patients were recruited following hospitalization from Tongji Hospital and the Institute of Hypertension (Wuhan, China) between September 2008 and September 2009. CAD was defined as one or more of the following diagnostic criteria: (*i*) >50% stenosis in at least one of the major segments of coronary arteries (the right coronary artery, left circumflex, or left anterior descending arteries) assessed by coronary angiography; (*ii*) World Health Organization criteria for elevated cardiac enzymes (troponin T, troponin I, creatine kinase-MB, aspartate aminotransferase, and glutamic pyruvic transaminase), typical ECG electrocardiogram change (Minnesota Code 1.1 or 1.2 in ECG), and clinical symptoms; or (*iii*) documented history of coronary artery bypass graft or percutaneous coronary intervention. Subjects with congenital heart disease, cardiomyopathy, valvular disease, and renal or hepatic disease were excluded from the study 22.

Ethnically and geographically matched controls were randomly recruited from the population by a house-to-house recruitment protocol. These controls were healthy, community-based residents. All control patients were free of cardiovascular diseases following the same exclusion criteria as cases. After coronary angiography examinations, individuals without any coronary stenosis were defined as healthy volunteers, while individuals with coronary stenosis but not reach the CAD diagnostic criteria [<50% stenosis in the major segments of coronary arteries (the right coronary artery, left circumflex, or left anterior descending arteries) assessed by coronary angiography] and also with risk factors of CAD were defined as CAD high-risk. Demographic data and other risk factors, including history of hypertension, diabetes, hyperlipidemia, smoking, and physical exercise, were collected by structured questionnaire. Hypertension was defined as systolic blood pressure >140 mmHg, diastolic blood pressure >90 mmHg, or current treatment with an antihypertensive drug. Diabetes was diagnosed by a fasting glucose level of >7.8 mmol/l and/or a glucose level of >11.1 mmol/l at 2 hrs after oral glucose challenge. Hyperlipidemia was defined as total plasma cholesterol level of >5.72 mmol/l or plasma triglyceride >1.70 mmol/l. Smoking was defined as a history of smoking >2 pack-years and/or smoking within the preceding 1 year. Body mass index (kg/m^2^) was calculated from the measurements of height and weight 23.

### miRNA microarray analysis

After informed consents were obtained, ten patients with CAD, 10 CAD high-risk patients and 10 healthy controls, respectively, at Tongji Hospital were recruited and 5 ml of peripheral blood was collected from each individual. All human research protocols were approved by the Clinical Research Committees of Tongji Medical College and were carried out according to the guidelines of the NIH. Plasma were isolated from peripheral blood by centrifugation, and mixed with TRIzol LS® reagent (Invitrogen, Life Technologies Corporation). Total RNA were harvested using TRIzol LS and RNeasy mini kit (QIAGEN, Shanghai, China) according to the manufacturer's instructions. After having passed RNA measurement on the Nanodrop instrument (Thermo Scientific, Guangzhou, China), the samples were labelled using the miRCURY™ Hy3™/Hy5™ Power labelling kit and hybridized on the miRCURY™ LNA Array (v.11.0; Exiqon Life Sciences, Vedbaek, Denmark). The samples were hybridized on a hybridization station following the instructions. Scanning was performed with the Axon GenePix 4000B microarray scanner (Molecular Devices, Inc., Silicon Valley, CA, USA). GenePix pro V6.0 was used to read the raw intensity of the image. The intensity of green signal is calculated after background subtraction and four replicated spots of each probe on the same slide have been calculated the median. We used Median Normalization Method to obtain ‘Normalized Data’, Normalized Data = (Foreground − Background)/median, the median is 50% quantile of microRNA intensity which is larger than 50 in all samples after background correction. The statistical significance of differentially expressed miRNA was analysed by *t*-test.

### Quantitative real-time PCR of miRNAs confirmation in additional population

Sixty additional individuals including 20 CAD patients, 20 CAD high-risk patients and 20 healthy controls were recruited, blood samples were drawn and total RNA was extracted using TRIzol LS and RNeasy mini kit. Total RNA from cultured cells or animal models were isolated using TRIzol (Invitrogen, Life Technologies Corporation). Two microgram of total RNA was reverse-transcribed using PrimeScript RT-PCR Kit (Takara Bio Co. Ltd., Dalian, China). qRT-PCR were performed with the Bulge-Loop™ miRNA qRT-PCR Detection Kit (Ribobio Co.) and SYBR® Premix Ex Taq™ (Takara Bio Co. Ltd.) according to the manufacturer's protocol with the Rotor-Gene 6000 system (Corbett Life Science, QIAGEN, Hilden, Germany). The U6 small RNA was used as the housekeeping small RNA reference gene. The relative gene expression was normalized to U6. Each reaction was performed in triplicate, and analysis was performed by the 2^−ΔΔCt^ method as described previously 24.

### Mice

The animals were housed in microisolator cages and fed *ad libitum* access to food and water. Twelve-week-old male ApoE^−/−^ mice and C57BL6 mice obtained from Vital River Laboratory Animal Technology Co., Ltd. (Beijing, China) were divided into six groups (*n* = 10 per group). Each group was treated *via* tail vein injection with different naked plasmids (negative treatment, pSilencer-random; pSilencer-anti-miR-320a; and pSilencer-miR-320a; 5 mg/kg every 4 weeks), respectively, while consuming a high-cholesterol diet containing 0.3% cholesterol and 21% (wt/wt) fat for 12 weeks. At killed, mice were fasted for 12 hrs before blood samples were collected. Anaesthetization was preformed with intraperitoneal injections of a xylazine (5 mg/kg) and ketamine (80 mg/kg) mixture, placed in a supine position before mice were killed. Tissue samples were collected and stored at −80°C.

### Biochemical parameters

Cholesterol, triglycerides, low-density lipoprotein (LDL) cholesterol, and high-density lipoprotein (HDL) cholesterol were measured on an AEROSET Clinical Chemistry System (Abbott Laboratories Santa Clara, CA, USA).

### Plasma analysis of atherosclerosis

Plasma IL-6, MCP-1, sICAM, pSelectin and TNF-α levels were determined *via* commercially available ELISA kits (R&D Systems, Minneapolis, MN, USA). Plasma fibrinogen levels were ascertained by ELISA kit (AssayPro, St. Charles, MO, USA).

### Nitric oxide release detection

Nitric oxide colorimetric assay kit was purchased from Biovision (Mountain View, CA, USA).

### Histology

After the animals were killed, organs were removed, fixed in 10% formalin, and embedded in paraffin. Four micrometre thick sections was prepared and stained with Masson trichrome for histological analysis. Snap-frozen fixed aortic rings embedded in optimal cutting temperature were sectioned, fixed in 4% PFA, and processed for Oil Red O staining according to standard protocols. MOMA-2 and α-actin expressions were analysed by Immunohistochemistry with DAB staining method. Images were acquired on an inverted microscope (Nikon Shanghai, China TE 2000) equipped with digital imaging, and were processed by Image-pro plus 6.0. For each slide, 5 HPF images were captured.

### Bioinformatics

miRNA target prediction was provided by different miRNA prediction programmes: MICROCOSM (http://www.ebi.ac.uk/enright-srv/microcosm/htdocs/targets/v5/), miRanda (http://www.microrna.org/microrna/home.do), TargetScan (http://www.targetscan.org/) and PICTAR-VERT (http://pictar.mdc-berlin.de/).

### Cell culture

2H-11 (mouse-derived endothelial cells) and HUVEC-CS (human-derived endothelial cells) were obtained from American Type Tissue Collection. 2H-11 cells were maintained in RPMI 1640 media supplemented with 10% FBS. HUVEC-CS cells were maintained in DMEM: Nutrient Mixture F-12 (DMEM/F12) media with 10% FBS. All cell cultures were maintained at 37°C in constant humidified incubator containing 95% air/5% CO_2_ atmosphere.

### Plasmid constructs

For the expressions of miR-320a and anti-miR-320a, two pairs of two complementary oligonucleotides were designed based on their sequences (Accession: MIMAT0000510), synthesized, annealed, and ligated into p*Silencer* 4.1-CMV neo vector (Ambion, ABI, Austin, TX, USA), respectively, according to the manufacturer's protocol. For the expressions of SP1 and SRF, the cDNAs were cloned into pcDNA3.1 (Invitrogen) expression vector separately. The nucleotide sequences of the constructed plasmids were confirmed by DNA sequencing analyses. Plasmid DNA was prepared for injection with E.Z.N.A Endo-free Plasmid Maxi Kit (Omega BioTek, Norcross, GA, USA), according to the manufacturers' protocol as described previously 25.

To construct pMIR-luciferase reporter plasmid, the 3′UTR fragment of SRF were inserted at the SacI and HindIII sites, into downstream of the luciferase gene in the pMIR-REPORT™ miRNA Expression Reporter Vector (Ambion, ABI) according to the manufacturer's protocol. Mutations were introduced using the Fast Mutagenesis System kit (TransGen, Beijing, China) according to the manufacturer's protocol. To construct pGL3-luciferase reporter plasmids, the 5′ fragments of hsa-mir-320a were inserted at the Mlu1 and Xho1 sites, into upstream of the luciferase gene in the pGL3.0 Vector (Promega Beijing, China) according to the manufacturer's protocol. The detail sequences were indicated in the figures. The nucleotide sequences of the constructed plasmids were confirmed by DNA sequencing analyses.

### RNA extraction and real-time PCR

Total RNAs were extracted using Trizol (Invitrogen) according to the manufacturer's instructions and reverse-transcribed by M-MLV First Strand cDNA Synthesis Kit (Invitrogen). The expression of miRNAs was measured by quantitative real-time PCR according to the manufacturer's protocol (Ribobio Co.) using Power SYBR Green PCR Master Mix (Invitrogen), and U6 small nuclear RNA was used as an internal normalized reference. Each reaction was performed in triplicate, and the results were analysed using 2^−ΔΔCt^ method.

### Western blotting

Proteins from cell lysates of cultured cells (20 μg) were separated by 10% SDS-PAGE electrophoresis and transferred to a PVDF membrane. After blocking in 5% non-fat milk, protein blots were incubated with specific antibodies followed by incubation with a peroxidase-conjugated secondary antibody in blocking buffer. The bands were visualized with the enhanced chemiluminescence method. Western blots were analysed densitometrically with ImageJ (National Institutes of Health software).

### Luciferase reporter assays

cDNA fragment corresponding to the entire 3′UTR of SRF was cloned into downstream Renilla luciferase open reading frame of the pMIR-REPORT™ miRNA Expression Reporter Vector (Ambion, ABI). 2H-11 cells and HUVEC cells were plated into 24-well plates (Costar Shanghai, China) and cotransfected with 200 ng of the different 3′UTR luciferase reporter vectors, 20 ng of a constitutively expressed firefly luciferase reporter vector, microRNA mimics (100 nM), microRNA random control (100 nM) or microRNA inhibitors (100 nM) utilizing Lipofectamine 2000 (Invitrogen) as described in the figure legends. Luciferase activity was measured using the Dual-Glo Luciferase Assay System (Promega). Renilla luciferase activity was normalized to the corresponding firefly luciferase activity and plotted as a percentage of the control (cells cotransfected with the corresponding concentration of microRNA random control). Experiments were performed in triplicate wells of a 24-well plate and repeated at least three times.

HUVEC cells were plated into 24-well plates (Costar) and cotransfected with 200 ng of the different 5′UTR luciferase reporter vectors (pGL3-), 20 ng of a constitutively expressed firefly luciferase reporter vector or different siRNA (100 nM) utilizing Lipofectamine 2000 (Invitrogen) as described in the figure legends. Luciferase activity was measured using the Dual-Glo Luciferase Assay System (Promega). Renilla luciferase activity was normalized to the corresponding firefly luciferase activity and plotted as a percentage of the control (cells cotransfected with the corresponding concentration of microRNA random control). Experiments were performed in triplicate wells of a 24-well plate and repeated at least three times.

### Transfection

Transfections were performed with Lipofectamine 2000 reagent (Invitrogen, Life Technologies Corporation) according to the manufacturer's instructions. Cells were plated in 96-well, 12-well or 6-well plates at a proper density. The cells were harvested for 24–48 hrs after transfection with plasmids or miRNAs/siRNAs. All control cells were treated with equal concentrations of a non-targeting control mimics sequence (miR Random) and a completely complementary sequence with the miRNA sequence (miR inhibitor), for use as controls for non-sequence-specific effects in miRNA experiments. Plasmid DNA for transient transfection was prepared with the TaKaRa MiniBEST Plasmid Purification Kit (Takara Bio Co. Ltd.).

### Cell cytotoxicity assay

Sulforhodamine B (SRB) was used to assess growth inhibition using a colorimetric assay which estimates cell number indirectly by staining total cellular protein with SRB as described previously 26. Briefly, cells were collected by trypsinization, counted, and plated at a density of 5000 cells/well in 96-well flat-bottomed microtiter plates (100 μl/well). At the end of each exposure, the medium was removed; cells were fixed with 20% (w/v) trichloroacetic acid at 4°C for 1 hr, and stained for 30 min. with 0.4% (w/v) SRB dissolved in 1% acetic acid. Wells were washed five times with 1% acetic acid and the protein-bound dye was solubilized with 10 mM Tris base (pH = 10). Each reaction was performed in triplicate. The optical density of cells was determined at a wavelength of 540 nm using a colorimetric plate reader.

### BrdU cell proliferation assay

To examine the effects of miR-320a on the growth of endothelial cells, we carried out BrdU cell proliferation assays. As described previously 27, the BrdU immunolabeling assay was performed with a non-isotopic immunoassay kit for the quantitation of bromodeoxyuridine incorporation into newly synthesized DNA of actively proliferating cells according to the manufacturer's instructions (Calbiochem, EMD Chemicals, Inc., Darmstadt, Germany). Briefly, transfected cells were incubated in a 96-well plastic plate using serum-free medium. BrdU was then added, and cells were cultured for another 8 hrs with tissue culture media. BrdU incorporated into the DNA was determined by measuring the absorbance at 450 nm on an ELISA plate reader. Each reaction was performed in triplicate.

### Determination of cell apoptosis by flow cytometry

After treatments with miRNAs as described above, the cells were harvested and resuspended in binding buffer, and incubated with FITC-conjugated Annexin V and propidium iodide (Annexin V-FITC Apoptosis Detection Kit, BD, San Jose, CA, USA) according to the manufacturer's protocol and then analysed with a FACStar-Plus flow cytometer (BD, Franklin Lakes, NJ, USA). To exclude necrotic cells, only the cells with Annexin V-positive and propidium iodide-negative staining were counted for early stages of apoptosis.

### Statistics

Data were presented as mean ± SEM (*n* was noted in the figure legends). The Wilcoxon test, the Student's *t*-test, and anova were performed, respectively, to determine statistical significance among treatment groups, as appropriate (depending on whether the data are normally distributed detected by Kolmogorov–Smirnov test. If data were normally distributed, then the Student's *t*-test and the anova were used, otherwise nonparametric statistics (Wilcoxon test) were used. In all cases, statistical significance was defined as *P* < 0.05.

## Results

### Microarray analysis of circulating miRNAs in patients and high-risk individuals of cardiovascular diseases

**Figure 1:**
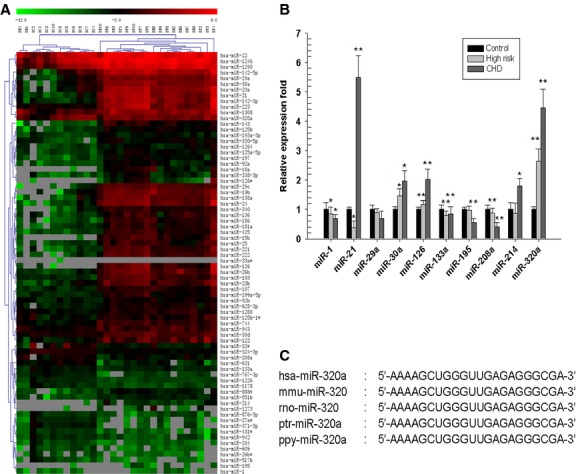
The Circulating miRNAs profile of CAD patients. (A) Partial heat map of up-regulated and down-regulated miRNA profiles. 10 CAD patients (DP1-DP10), 10 patients with CAD high-risk (DR1-DR10) and 10 healthy control patients (DC1-DC10) were included. Red indicates high expression and green indicates low expression. (B) Real-time PCR analysis of the expression of miRNAs. A second group of 20 CAD patients, 20 patients with CAD high risk and 20 healthy control patients were recruited, and 10 candidate miRNAs were analysed. *P < 0.05 *versus* control, **P < 0.01 *versus* control, data are representative of three experiments (*n* = 3). (C) Comparison of the miR-320 sequences among different species.

Disease-associated miRNAs can often be detected in circulation by sensitive methods. We used microarray analysis to determine the circulating miRNA profiles in 10 CAD patients, 10 patients with CAD high-risk factors and 10 healthy individuals (Table S1). A subset of miRNAs in human peripheral plasma differentially expressed in CAD patients and high-risk patients (Fig.1A and Fig. S1), including 57 miRNAs with increased expression and 18 miRNAs with decreased expression (Table1). We validated 10 of the differentially expressed miRNAs by bulge-loop miRNA real-time PCR assay in a second cohort (Table S2). Seven miRNAs (miR-21, miR-30a, miR-126, miR-133a, miR-195, miR-208a and miR-320a) were confirmed to be differentially expressed between CAD and control samples (Fig.1B). Further, we measured 10 more circulating miRNAs expression in a third cohort (Table S3), and most of the results were consistent with previous reports (Fig. S2). Sequence alignment revealed that one of these candidates miR-320a is encoded within the intergenic region of chromosome 8. The sequence coding for miR-320a is highly conserved in mammals (Fig.1C), which prompted us to choose miR-320a for further study.

**Table 1:** Selectively up-regulated or down-regulated miRNAs are consistent in CAD patients and high-risk patients determined by fold changes

miRNA ID	High-risk patients		CAD patients	
	Fold change	*P*-value	Fold change	*P*-value
hsa-miR-142-3p	48.9755	0.0036	176.8939	0.0273
hsa-miR-126	24.3953	0.0186	173.2294	0.0435
hsa-miR-126^*^	14.0498	<0.0001	162.5304	<0.0001
hsa-miR-320a	76.5025	0.0390	162.4763	0.0462
hsa-miR-15b	37.5948	<0.0001	142.3075	<0.0001
hsa-miR-21	35.4103	0.0049	127.4212	0.0008
hsa-miR-24	23.6256	0.0012	92.2378	0.0425
hsa-miR-18a	73.0136	<0.0001	87.9023	<0.0001
hsa-miR-26b	21.6182	0.0338	57.1140	0.0311
hsa-miR-223	7.6881	0.0135	52.8067	0.0059
hsa-miR-33a	20.8649	0.0130	50.6467	0.0033
hsa-miR-222	19.9843	0.0029	46.6978	0.0001
hsa-miR-23a	12.0997	0.0083	42.4228	0.0064
hsa-miR-221	10.7487	0.0037	36.5754	0.0050
hsa-miR-186	13.9774	0.0062	26.4288	0.0044
hsa-miR-130a	9.3914	0.0080	25.4403	0.0015
hsa-miR-103	10.2418	0.0014	24.4025	0.0004
hsa-miR-23b	5.3145	0.0029	24.0128	0.0013
hsa-miR-142-5p	13.1173	0.0031	23.9530	0.0031
hsa-miR-25	7.1326	0.0299	23.9375	<0.0001
hsa-miR-29c	20.6553	0.0035	23.5149	<0.0001
hsa-miR-19b	19.9748	0.0318	23.4687	0.0001
hsa-miR-425	10.2032	0.0117	23.1517	0.0019
hsa-miR-143	11.3411	0.0293	23.1220	0.0009
hsa-miR-29a	12.7221	<0.0001	22.7786	0.0004
hsa-miR-30a	10.2149	0.0040	21.2178	<0.0001
hsa-miR-181a	8.2078	0.0069	20.7860	0.0376
hsa-miR-122	8.3698	0.0317	17.4977	0.0197
hsa-miR-136	9.5445	0.0050	15.1647	0.0006
has-miR-214	4.4856	<0.0001	13.9375	<0.0001
hsa-miR-1273	40.3892	<0.0001	10.8984	<0.0001
hsa-miR-340	5.4088	0.0028	9.3606	0.0003
hsa-miR-199a-5p	2.6213	0.0406	8.7199	0.0049
hsa-miR-107	4.5310	0.0055	8.2907	0.0047
hsa-miR-92a	4.2103	0.0319	7.2702	<0.0001
hsa-miR-1308	7.3035	0.0008	7.1501	0.0067
hsa-miR-1290	4.7888	0.0034	6.8817	<0.0001
hsa-miR-197	3.9302	0.0046	6.6063	0.0001
hsa-miR-338-3p	2.9401	0.0314	6.4546	0.0117
hsa-miR-195	1.6340	<0.0001	6.2436	<0.0001
hsa-miR-125b	3.6804	0.0052	5.8632	<0.0001
hsa-miR-125a-5p	3.2639	0.0004	5.3843	0.0002
hsa-miR-888^*^	3.3083	0.0004	5.1987	0.0006
hsa-miR-1264	3.1297	0.0073	4.6689	0.0021
hsa-miR-744	4.0746	0.0006	4.6290	0.0001
hsa-miR-330-5p	5.4841	<0.0001	4.5780	0.0004
hsa-miR-1246	2.8165	0.0117	4.3614	0.0001
hsa-miR-30d	2.3328	0.0378	3.8085	<0.0001
hsa-miR-628-3p	3.6025	0.0003	3.6516	0.0006
hsa-miR-551b	2.8577	0.0003	3.3751	0.0006
hsa-miR-22	2.5051	0.0137	2.9987	0.0009
hsa-miR-125b-1^*^	2.0962	0.0296	2.9517	0.0189
hsa-miR-92b	2.7240	0.0047	2.4624	<0.0001
hsa-miR-193a-3p	2.5077	0.0068	2.2892	0.0039
hsa-miR-943	4.5303	0.0001	2.2722	0.0076
hsa-miR-1280	3.1999	0.0006	2.1144	0.0001
hsa-miR-1	1.0634	0.8545	0.9599	0.9181
hsa-miR-133a	0.4710	0.0039	0.6394	0.0427
hsa-miR-208a	1.0634	0.6138	0.5498	0.0079
hsa-miR-1226	0.3946	0.0010	0.4771	0.0040
hsa-miR-767-3p	0.3865	<0.0001	0.4249	0.0001
hsa-miR-324-3p	0.3580	<0.0001	0.4131	<0.0001
hsa-miR-631	0.3827	0.0012	0.3776	0.0003
hsa-miR-1178	0.4021	0.0025	0.3707	0.0015
hsa-miR-942	0.3916	0.0209	0.3693	0.0123
hsa-miR-204	0.2707	0.0046	0.3344	0.0187
hsa-miR-431^*^	0.2589	0.0016	0.3271	0.0023
hsa-miR-609	0.3212	0.0355	0.2819	0.0272
hsa-miR-876-3p	0.4845	0.0482	0.2668	0.0132
hsa-miR-26b^*^	0.2973	0.0229	0.2336	0.0335
hsa-miR-517b	0.2874	0.0359	0.1532	0.0387
hsa-miR-27a^*^	0.2980	0.0177	0.1435	0.0023
hsa-miR-32^*^	0.1498	0.0496	0.1244	0.0421
hsa-miR-371-3p	0.2742	0.0097	0.0994	0.0041

### MiR-320a promotes adverse serum lipid profiles and atherogenesis in wild-type and ApoE^−/−^ mice

**Figure 2:**
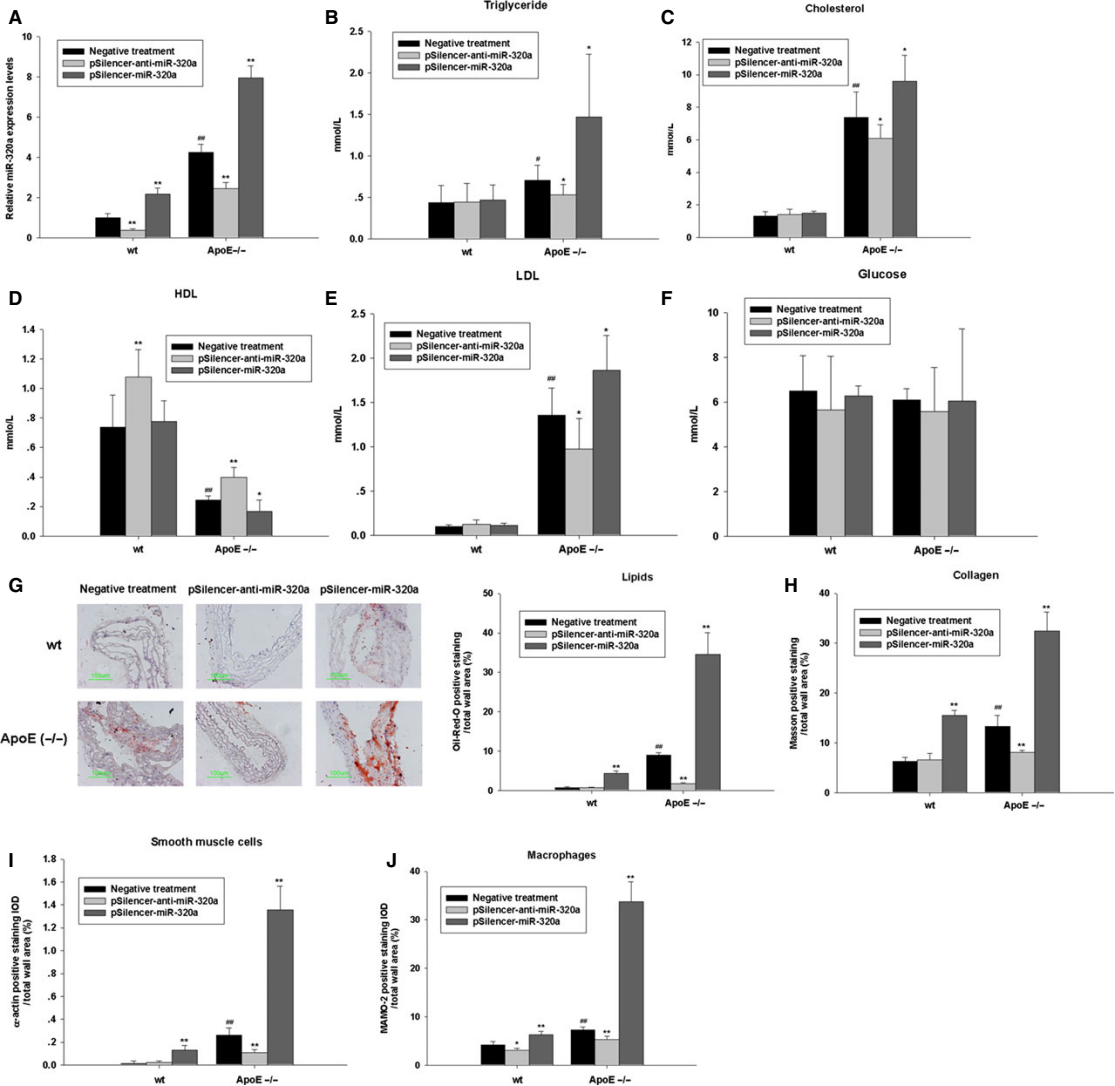
Effects of miR-320a on lipids metabolism and plaque formation in wild-type and ApoE^−/−^ mice. (A) Different plasmids were injected *via* tail vein at a dose of 5 mg/kg, every 4 weeks. The levels of miR-320a in aorta were determined at 12 weeks by real-time PCR assays, which showed that the expression of mature miR-320a were significantly increased in ApoE^−/−^ mice compared with wild-type C57BL6 mice, and pSilencer-anti-miR-320a treatment reduced the mature miR-320a in both wild-type mice and ApoE^−/−^ mice, pSilencer-miR-320a treatment elevated mature miR-320a expression in both wild-type mice and ApoE^−/−^ mice. (B–F) Fasting triglycerides, cholesterol, HDL, LDL and glucose levels of wild-type and ApoE^−/−^ mice after plasmids injection for 12 weeks. (G–J) Overexpression of miR-320a promotes atherosclerotic plaque formation in ApoE^−/−^ mice as determined by histological staining and immunohistochemistry. (G) Oil red O–stained frozen section of aortic sinus from mice fed on a high-cholesterol diet with different treatments. Sections of aortic arches of mice treated as described above were analysed for: Masson's trichrome stain, with collagen staining blue (H); α-actin immunohistochemistry to identify vascular smooth muscle cells (I); MaMo-2 immunohistochemistry to visualize plaque area occupied by macrophages (J). Data are expressed as mean ± SE (*n* = 10), ^#^*P* < 0.05 *versus* wild-type with negative treatment, ^##^*P* < 0.01 *versus* wild-type with negative treatment, *P < 0.05 *versus* same genotype with negative treatment, **P < 0.01 *versus* same genotype with negative treatment.

To test the effects of miR-320a on serum lipid profiles, plasmids carrying negative control sequence (negative treatment), miR-320a sense or antisense sequences (pSilencer-miR-320a and pSilencer-anti-miR-320a) were intravenously administrated to high-fat fed ApoE^−/−^ mice and wild-type mice every 4 weeks for 12 weeks. The delivery efficiency was shown in Figure S3A. As shown in Figure2A and Figure S3B, treating mice with pSilencer-miR-320a markedly increased miR-320a expression while treating with pSilencer-anti-miR-320a significantly suppressed miR-320a expression in aorta and plasma. To examine the effects of miR-320a on plasma lipid levels, we measured plasma levels of TG, TC, LDL, and HDL as well as plasma glucose level at the end-point of treatments (Fig.2B–F). In both wild-type and ApoE^−/−^ mice, miR-320a overexpression resulted in significant increase in TC, TG and LDL and significant decrease in HDL level. However, the glucose levels were not affected. Oil Red O staining was used to highlight intralesional lipid deposition. ApoE^−/−^ mice received pSilencer-miR-320a treatment developed significantly larger atherosclerotic lesions throughout the aorta comparing to the controls (Fig.2G). Interestingly, wild-type mice treated with pSilencer-miR-320a also developed atherosclerotic lesions (Fig.2G). MiR-320a overexpression also increased vascular collagen deposition in ApoE^−/−^ mice as shown by Masson trichrome staining (Fig.2H). Furthermore, analysis of plaque composition revealed a significant increase in smooth muscle cell content, whereas macrophage content also tended to be increased in the miR-320a treated ApoE^−/−^ mice, indicated by α-actin staining and MAMO-2 staining (Fig.2I and J, respectively). These results demonstrate that miR-320a adversely regulates lipid metabolism and therefore contributes to development of atherosclerosis and CAD.

### Effects of miR-320a on serum inflammatory cytokines and endothelium cell function in wild-type and ApoE^−/−^ mice

**Figure 3:**
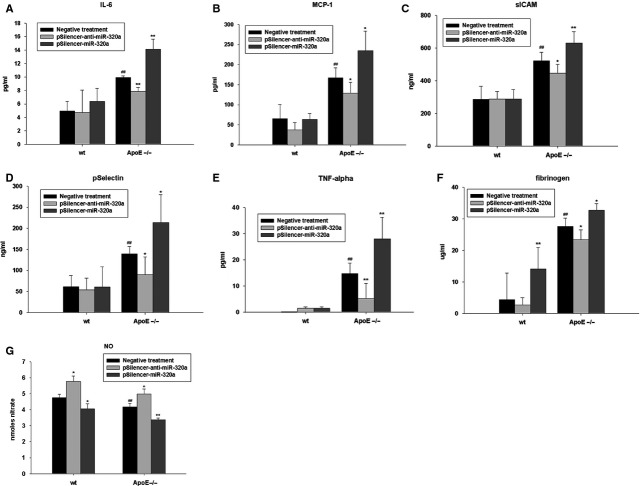
MiR-320a affects atherosclerosis in ApoE^−/−^ mice. Quantitative ELISA detection of IL-6 (A), MCP-1 (B), ICAM (C), selectin (D), TNF-α (E), fibrinogen (F) and nitric oxide (G) were measured from plasma of wild-type mice and ApoE^−/−^ mice treated with different plasmids. Data are expressed as mean ± SE (*n* = 10), ^#^*P* < 0.05 *versus* wild-type with negative treatment, ^##^*P* < 0.01 *versus* wild-type with negative treatment, *P < 0.05 *versus* same genotype with negative treatment, **P < 0.01 *versus* same genotype with negative treatment.

We next investigated the effects of miR-320a on the levels of plasma proatherosclerotic proteins. Hereto, we measured plasma levels of IL-6, MCP-1, sICAM, pSelectin, TNF-α and fibrinogen in ApoE^−/−^ mice and wild-type mice (Fig.3A–F). The basal plasma levels of the inflammatory markers were significantly increased in ApoE^−/−^ mice compared to the wild-type mice. Significantly, pSilencer-miR-320a injection resulted in marked elevation of proinflammatory cytokines (Fig.3A–F). Conversely, pSilencer-anti-miR-320a treatment resulted in the dampening of the proinflammatory response. We next examined the effects of miR-320a on nitric oxide production, an important indicator of endothelium cell function (Fig.3G). It was observed that aortic release of nitric oxide was decreased in ApoE^−/−^ mice compared with wild-type mice; pSilencer-anti-miR-320a treatment stimulated nitric oxide release and pSilencer-miR-320a treatment repressed nitric oxide release obviously. These results strongly indicate miR-320a promotes proinflammatory reactions, which greatly contribute to endothelia dysfunction, key components of atherosclerosis genesis and development.

### SRF is a physiological target of MiR-320a

**Figure 4:**
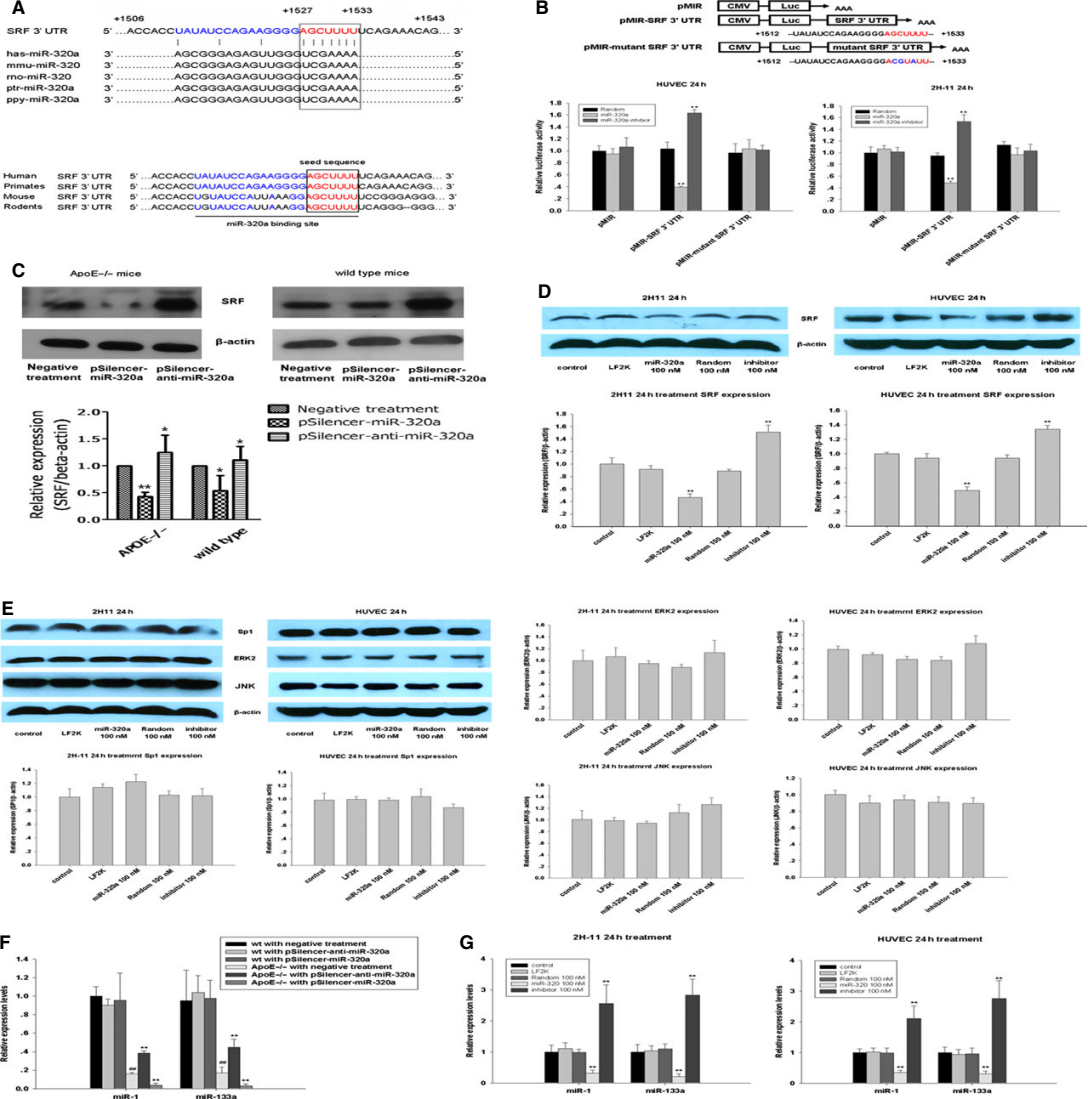
SRF is a target of miR-320. (A) Upper: sequence alignment of miR-320a among different species and 3′UTR of human SRF. The 3′ end of predicted binding site (characters in red) in human SRF was completely complementary with the 5′ end of miR-320a, where the crucial seed regions (characters in blue) are located; Lower: the putative miR-320a binding sites within the SRF 3′UTR are conserved among mammalian species. (B) Dual luciferase activity assay in 2H-11 cells and HUVEC cells cotransfected with various plasmids (0.4 μg/ml) (pMIR: control reporter plasmid, pMIR-3′UTR SRF: reporter plasmid containing SRF 3′UTR, pMIR-mutant 3′UTR SRF: reporter plasmid containing mutant SRF 3′UTR) and miR-320a random (100 nM), miR-320a mimics (100 nM), and miR-320ainhibitor (100 nM). The results showed that miR-320a inhibited luciferase activity, compared with controls. The seed regions were characters in red and the mutation were characters in blue, *n* = 3. (C) Protein level expression of SRF in aorta of ApoE^−/−^ mice and wild-type mice with various treatments, *n* = 10. (D) Total cellular proteins from treated 2H-11 cells and HUVEC cells were probed using a SRF antibody, *n* = 3. (E) Sp1, ERK2 and JNK, which involve in the SRF regulation pathway, were detected by immunoblots in 2H-11 cells and HUVEC cells, *n* = 3. (F) The expression of miR-1 and miR-133a in aorta of treated mice were determined by real-time PCR, *n* = 10. (G) The *in vitro* detection of miR-1 and miR-133a expression under miR-320a (100 nM) treatment by real-time PCR assays, *n* = 3. Data are expressed as relative percentage compared with control, *P < 0.05 *versus* control, **P < 0.01 *versus* control, ^##^*P* < 0.01 *versus* wild-type with negative treatment.

To look into the mechanisms of miR-320a in promoting atherosclerosis progress, we first identified the putative targets of miR-320a using computational predictions as described at MICROCOSM, miRanda, TargetScan and Pic Tar. Considering the major function of miRNAs is to regulate protein expression, we analysed the profile of diseases and pathways that predicted protein targets of miR-320a may involved in (Table2). Since hsa-miR-320 participates in cardiac ischemia/reperfusion injury and lipid/glucose metabolism 7,28,29. We focused on one of the potential targets (SRF/c-fos serum response element-binding transcription factor), since it is crucial in cardiovascular physiology and its high probability to be a genuine target of miR-320a as revealed by bioinformatics analysis. The complimentary seed sequences of hsa-miR-320a were located at 1527–1533 bp of human SRF 3′UTR (Fig.4A). The seed sequences of SRF 3′UTR binding site are highly conserved among human, primates, mouse and rodents (Fig.4A).

**Table 2:** Signal pathways of the predicted targets miR-320a may involve

Pathway name	Frequency in the target gene group	Frequency in all genes	*P*-value	Fold ratio
Multi-step regulation of transcription by Pitx2	3 of 87 (0.0345%)	16 of 5525 (0.0029%)	0.0017	11.9073
NF-kB signalling pathway	2 of 87 (0.023%)	23 of 5525 (0.0042%)	0.0448	5.5222
Nuclear receptors coordinate the activities of chromatin remodelling complexes and coactivators to fa	2 of 87 (0.023%)	15 of 5525 (0.0027%)	0.021	8.4674
p38 MAPK signalling pathway	3 of 87 (0.0345%)	39 of 5525 (0.0071%)	0.0198	4.8851
Phosphoinositides and their downstream targets	3 of 87 (0.0345%)	39 of 5525 (0.0071%)	0.0198	4.8851
RB tumour suppressor/checkpoint signalling in response to DNA damage	3 of 87 (0.0345%)	13 of 5525 (0.0024%)	0.0009	14.6552
Regulation of cell cycle progression by Plk3	2 of 87 (0.023%)	14 of 5525 (0.0025%)	0.0185	9.0722
Regulation of PGC-1a	2 of 87 (0.023%)	24 of 5525 (0.0043%)	0.0481	5.2921
RNA polymerase III transcription	2 of 87 (0.023%)	8 of 5525 (0.0014%)	0.0063	15.8764
Role of Erk5 in neuronal survival	2 of 87 (0.023%)	18 of 5525 (0.0033%)	0.0293	7.0562
Role of nicotinic acetylcholine receptors in the regulation of apoptosis	2 of 87 (0.023%)	16 of 5525 (0.0029%)	0.0237	7.9382
Signal-dependent regulation of myogenesis by corepressor MITR	3 of 87 (0.0345%)	10 of 5525 (0.0018%)	0.0004	19.0517
SODD/TNFR1 signaling pathway	2 of 87 (0.023%)	10 of 5525 (0.0018%)	0.0097	12.7011
Sonic hedgehog (SHH) receptor Ptc1 regulates cell cycle	2 of 87 (0.023%)	11 of 5525 (0.002%)	0.0117	11.5465
Spliceosomal assembly	2 of 87 (0.023%)	14 of 5525 (0.0025%)	0.0185	9.0722
Sumoylation by RanBP2 regulates transcriptional repression	2 of 87 (0.023%)	10 of 5525 (0.0018%)	0.0097	12.7011
Trefoil factors initiate mucosal healing	3 of 87 (0.0345%)	24 of 5525 (0.0043%)	0.0055	7.9382
Wnt_signaling	4 of 87 (0.046%)	61 of 5525 (0.011%)	0.0126	4.1643

The specific nodes of functional annotation categories that miR-320a were statistically enriched in. Pathway name shows the pathways that the predicted targets miR-320a may involved in; Frequency in target gene group shows the number of GO associations belonging to the pathway; Frequency in all genes shows the background distribution from all human genes GO associations.

Vectors carrying luciferase reporter gene without the SRF 3′UTR, with the SRF 3′UTR or with the mutant SRF 3′UTR were cotransfected with miR-320a mimics, random or inhibitor. MiR-320a mimics resulted in significant decrease in luciferase activity while miR-320a inhibitor slightly increased luciferase activity (Fig.4B). No change in luciferase activity was observed when miR-320a random control was used or when miR-320a mimics was co-transfected with the control vector without the SRF 3′UTR or with the mutant SRF 3′UTR. These observations strongly suggest that SRF is a physiological target of miR-320a. To provide further evidence, we examined the effects of miR-320a on SRF expression *in vivo* and *in vitro* by western blot. The results showed that the level of SRF expression was significantly reduced in ApoE^−/−^ and wild-type mice treated with pSilencer-miR-320a comparing to the controls; whereas knockdown of endogenous miR-320a by pSilencer-anti-miR-320a injection increased the SRF expression (Fig.4C). In *in vitro* assays, we transfected 2H-11 cells and HUVEC cells with miR-320a mimics, random control and inhibitor. MiR-320a transfection significantly reduced SRF expression (Fig.4D). The combined data show that miR-320a negatively regulates the SRF expression. It is reported that SRF may be induced by other genes, such as Sp1, ERK2 and JNK 19,30. However, miR-320a did not show any effects on the expression of Sp1, ERK2 and JNK in endothelium cells, indicating that miR-320a directly regulates SRF rather than *via* indirect pathways (Fig.4E). Interestingly, the expression miR-1 and miR-133a, miRNAs regulated by SRF 21, were significantly decreased by miR-320a transfection *in vivo* and *in vitro* (Fig.4F and G).

Furthermore, we investigated the expression and function of miR-320a in liver. MiR-320a treatment depressed SRF expression and its downstream factors in liver from ApoE^−/−^ mice: LXR, an important nuclear receptor target of the cholesterol metabolites; sterol response element-binding protein 2 (SREBP2), which promotes the transcription of the LDL receptor (Fig. S4A). And as a result, the free fatty acid levels in liver tissue were increased and anti-miR-320a treatment reversed the effects (Fig. S4B). And the expressions of miR-320a in liver were shown in Figure S4C. HE staining suggested that the primary effect of miR-320a on adverse lipid profiles were not from the adverse effect of hepatic injury due to the method itself (Fig. S4D).

Taken together, these data indicate that SRF is a physiological target of miR-320a.

### MiR-320a inhibits endothelia cell proliferation and induces cell apoptosis *via* SRF

**Figure 5:**
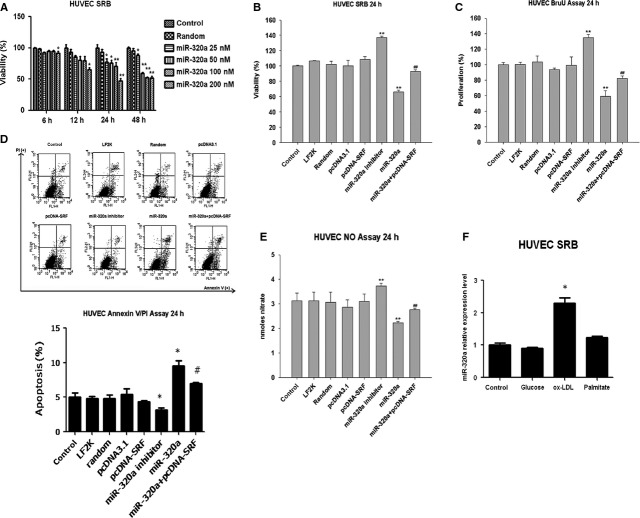
Effects of miR-320a on cell viability and function in cultured endothelium cells. (A) Time-dependent and dose-dependent effects of miR-320a on cell viability as determined by SRB assays. (B) Effects of miR-320a mimics (100 nM) and pcDNA-SRF (0.4 μg/ml) on the survival of human endothelium cells (HUVEC) as determined by SRB assays (24 hrs). (C) Effects of miR-320a mimics (100 nM) and pcDNA-SRF (0.4 μg/ml) on the cell proliferation of human endothelium cells (HUVEC) as determined by BrdU incorporation assays (24 hrs). (D) Effects of miR-320a mimics (100 nM) and pcDNA-SRF (0.4 μg/ml) on apoptosis of human endothelium cells (HUVEC) as determined by Annexin V/PI flow cytometric analysis (24 hrs). (E) Effects of miR-320a mimics (100 nM) and pcDNA-SRF (0.4 μg/ml) on nitric oxide release. (F) Effects of pro-atherosclerotic factors on miR-320a expression (Glucose: 25 mM; ox-LDL: 50 μg/ml; Palmitate: 200 μM). LF2K refers to lipofectamine 2000, *n* = 3. Data are expressed as relative percentage compared with normal control, *P < 0.05 *versus* control, **P < 0.01 *versus* control, ^##^*P* < 0.01 *versus* miR-320a.

Both gain-of-function and loss-of-function approaches were employed to explore the role of miR-320a and SRF in cultured endothelial cells. We constructed SRF expression plasmid (pcDNA-SRF), synthesized hsa-miR-320a mimics, random control and inhibitor (referred as miR-320a, random and inhibitor, respectively) and transfected cultured endothelial cells as indicated in the figure legends. Cell viability analysis showed that addition of miR-320a decreased the number of viable cells in a time- and dose-dependent manner, while treatment of miR-320a inhibitor increased the number of viable cells as determined by the SRB assay (Fig.5A and B). We then tested the effects of miR-320a on cell proliferation and apoptosis by using BrdU incorporation assay and Annexin V/PI staining assay, respectively. Consistent with the results of SRB assays, overexpression of miR-320a inhibited cell proliferation and induced apoptosis, while knocking down endogenous miR-320a by miR-320a inhibitor showed opposite effects (Fig.5C and D). Moreover, miR-320a significantly suppressed nitric oxide release in HUVEC cells, while miR-320a inhibitor directly stimulated nitric oxide production (Fig.5E). The most important of all, overexpression of SRF reversed the effects of miR-320a. Interestingly, ox-LDL, which is also the most important proatherosclerotic factor, increased miR-320a significantly, other than glucose or palmitate (Fig.5F). Taken together, these data suggest that miR-320a plays important roles in endothelial cell dysfunction, a key event for atherogenesis *via* SRF.

### SP1 regulates miR-320a expression in endothelial cells

**Figure 6:**
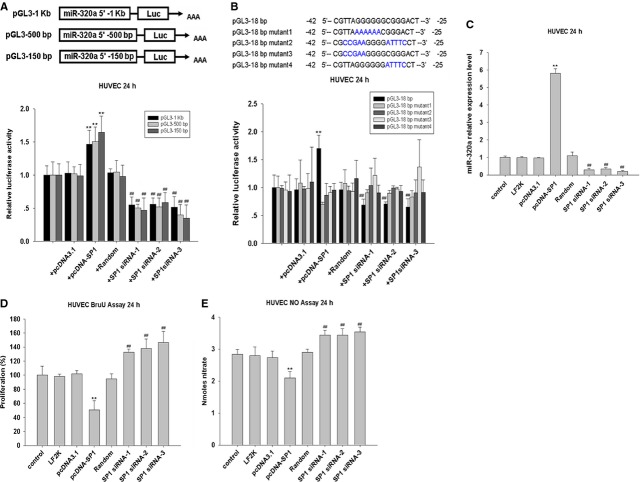
SP1 regulates miR-320a expression in endothelial cells. (A) Dual luciferase activity assay in HUVEC cells cotransfected with pGL3 plasmids contain deferent length of 5′ flank of hsa-miR-320a (0.4 μg/ml), pcDNA-SP1 (0.4 μg/ml) and SP1-siRNAs (100 nM), *n* = 3. (B) Dual luciferase activity assay in HUVEC cells cotransfected with pGL3 plasmids contain deferent mutation of SP1 binding site in 5′ flank of hsa-miR-320a (0.4 μg/ml), pcDNA-SP1 (0.4 μg/ml) and SP1-siRNAs (100 nM), *n* = 3. (C) The *in vitro* detection of miR-320a expression under SP1 treatments (plasmid: 0.4 μg/ml; siRNA: 100 nM) by real-time PCR assays, *n* = 3. (D) The effect of SP1 treatments (plasmid: 0.4 μg/ml; siRNA: 100 nM) on proliferation as detected by ELISA assays, *n* = 3. (E) The *in vitro* detection of nitric oxide release under SP1 treatments (plasmid: 0.4 μg/ml; siRNA: 100 nM) by ELISA assays, *n* = 3. Data are expressed as relative percentage compared with normal control, *P < 0.05 *versus* control, **P < 0.01 *versus* control, ^##^*P* < 0.01 *versus* Random.

How might miR-320a expression be upregulated in atherosclerosis? We analysed the promoter region of hsa-mir-320a and observed that it contains several binding sites for the transcription factor SP1, which contributes to lipid metabolism and inflammation 31. To analyse the activity of the putative hsa-mir-320a gene promoter, three plasmids pGL3-1K (−885/+195), pGL3-500bp (−427/+96) and pGL3-150bp (−152/+96) were constructed by placing different length of the putative promoter region and the 5′-end of the gene in front of the reporter coding sequence (Fig.6A), and were cotransfected with SP1 expression plasmid (pcDNA-SP1) and SP1 specific siRNAs into HUVEC cells. Interestingly, fluorescence signal could be enhanced in the cells transfected with pcDNA-SP1, while SP1 siRNA showed the opposite effects. Moreover, fluorescence signal could be observed in the cells transfected with pGL3-150bp similarly as the cells transfected with pGL3-1Kb (Fig.6A). To assess the importance of the potential 18bp SP1 binding sites within the −150bp fragment, mutations were introduced into the binding sites of transcription factor SP1 in the plasmid pGL3 (Fig.6B). As expected, SP1 overexpression induced hsa-mir-320a promoter activity, while down-regulation of SP1 suppressed hsa-mir-320a promoter activity, and these effects were reversed by mutations introduced into the 18bp region (Fig.6B). In addition, SP1 overexpression increased miR-320a level, and knockdown of SP1 reduced miR-320a level (Fig.6C). These data suggest that SP1 is involved in the regulation of miR-320a expression.

We next explored whether SP1 is able to regulate endothelial cell function. SP1 overexpression in endothelium inhibited proliferation, whereas knockdown of SP1 promoted proliferation (Fig.6D). Moreover, overexpressed SP1 reduced nitric oxide release, while down-regulation of SP1 improved nitric oxide release in HUVEC cells (Fig.6E). Thus, SP1 seems to participate in the regulation of miR-320a, although SP1 may also target other factors involved in endothelial cell function.

## Discussion

Circulating miRNAs may be useful plasma biomarkers for the diagnosis, prognosis and therapy of various diseases 32,33. The biological function of differentially expressed circulating miRNAs and their correlation with diseases are still not fully understood. Here, we show that miR-320a is markedly elevated in both CAD patients and high risk individuals. Based on these findings, a series of experiments were conducted to evaluate the potential role of miR-320 in the development of atherosclerosis. *In vitro*, miR-320a was shown to inhibit endothelial cells proliferation and induced apoptosis of endothelial cells. *In vivo*, a striking regulation of lipid profiles was detected and atherosclerotic lesion formation was regulated by systemic injection of miR-320a or miR-320a inhibitor encoding plasmid in ApoE^−/−^ mice. Moreover, induced by SP1, miR-320a downregulated SRF, a key endothelial cell regulator essential for VGEF induced cell signalling and angiogenesis, and contributed to endothelial dysfunction.

Abnormal expression profiles of miRNAs have been identified in plasma of patients with heart failure and myocardial injury 33,34. However, the functions of these circulating miRNAs are still lacking. We compared CAD cases not only with the healthy controls but also with the patients with high risks for CAD. This enabled us to distinguish miRNAs that are associated with atherosclerosis progression.

In addition to miR-320a, we found a group miRNAs which are differentially expressed in CAD patients, among which miRNAs, miR-21, miR-30a, miR-126, and miR-133a were reported to be up-regulated and miR-208a and miR-320a to be downregulated in infarcted myocardium 35,36.

Recently, it has been suggested that the overwhelming majority of the nuclease-resistant extracellular miRNA in plasma and cell culture media is associated with microparticles, exosomes or Ago2 protein, indicating that circulating miRNAs in blood plasma/serum as potential biomarkers for early disease diagnoses 37. However, the mechanism by which circulating miRNAs are released into the circulation is unclear. And diseases that underwent similar pathophysiology may exhibit similar miRNA expression profiles, makes it hard to distinguish the sub-clinical pathological sources of miRNAs.

It has long been realized that the trigger event in atherogenesis is the loss of endothelial integrity, which leads to the intimal lipoprotein deposition, inflammation, and dysfunction of vasodilation 38. SRF involves in regulating multiple cell functions including cellular proliferation, regulation of myogenesis 39, cell migration 40, cell adhesion 41, cytoskeletal assembly and organization 42, and extracellular matrix production 43. It was also reported that SRF mediates glucose response *via* its binding sites in LXRB gene promoter 44. The liver X receptor (LXR) pathway can act as “cholesterol sensors” and in collaborating with the SREBPs, regulates reverse cholesterol transport, cholesterol conversion into bile acid, and intestinal cholesterol absorption 45. Interestingly, recent studies have shown that SRF regulates the expression of miR-1 and miR-133a, miRNAs important for cardiac and skeletal muscles 46,47. We detected the expressions of miR-1 and miR-133a by real-time PCR in aorta of miR-320a treated mice and endothelium cells treated with miR-320a. Indeed, the expression of miR-1 and miR-133a were regulated by miR-320a. We speculate miR-1 and miR-133a are indirect targets of miR-320a downstream of SRF. MiR-1, miR-133a and other targets of SRF may contribute to the development of atherosclerosis and CAD.

Intergenic miRNAs are independent transcription units, with their own transcriptional regulatory elements. And 60% of miRNAs have more than half of their TF sites within 1 kb 48. As we identified, SP1, a transcription factor, contributes to lipid metabolism and inflammation, regulate miR-320a expression directly.

Taken together, in current study, we determined the circulatory level of miR-320a in coronary heart disease patients and investigated its role in lipid metabolism and atherosclerosis formation utilizing *in vivo* and *in vitro* experimental models. It was observed that circulating miRNA-320a was highly expressed in CAD patients. Further, overexpression of miR-320a resulted in significant increase in levels of plasma lipid and serum inflammatory cytokines. Utilizing ApoE^−/−^ mice, it was found that miR-320a expression attenuates endothelium cell function and promotes atherogenesis. Bioinformatics analysis identified serum response factor as a potential target for miR-320a, which was validated by luciferase reporter activity assay and Western-blot both *in vitro* and *in vivo*. Finally, it was demonstrated that miR-320a expression inhibits human-derived endothelium cell proliferation and induces apoptosis.

Still, there are some limitations in our study. Although the plasma miR-320a levels were significantly higher in the high risk and CAD patients, there is very little difference in the lipid profile values among healthy volunteers, high-risk patients, and CAD patients. Indicating that among these risk factors, each may contributes differently to the circulating miRNAs profile. And there may be cross-influences among them. Validation of the high-risk group showed 50% validation and 20% opposite significant changes. This may be induced by varieties in type and degree of risk factors among each individual. The association between individual risk factor and individual miRNA expression requires a more rigorous design and complex analysis. And follow-up studies investigating whether these risk-related miRNAs were associated with the development of CAD need to be conducted. MiR-320a distribution in lesion should also be identified in the future research.

In conclusion, our data provide evidence that overexpression of miR-320a is a key risk factor for atherosclerosis. Our data reveal links among SP1, miR-320a, SRF and miR-1/miR-133a in endothelial dysfunction in atherosclerosis. These findings may provide an entry point for studying the role of miRNAs and their targets in the initiation and progress of atherosclerosis and cardiovascular diseases. MiR-320a may serve as a novel biomarker and therapeutic target for atherosclerosis and CAD.

## Supporting Information

**Data S1** Scatter plot analysis of the microarray profile of circulating miRNAs.
