# Supplementary material for: MiR-320a contributes to atherogenesis by augmenting multiple risk factors and down-regulating SRF
Source: J Cell Mol Med. 2015 Feb 27;19(5):970–85. doi: 10.1111/jcmm.12483 (PMC4420600; doi:10.1111/jcmm.12483)
Supplement: Supplementary file 1 [file jcmm0019-0970-sd1.doc]

**Online supplemental material**

Fig. S1 is scatter plot analysis of the microarray profile of circulating miRNAs. (A) Group DC vs. group DP; (B) Group DC vs. group DP; (C) Group DR vs. group DP. Fig. S2 is real-time PCR analysis of the expression of circulating miRNAs. A third group of 50 CAD patients and 50 healthy control people were recruited, and 12 candidate miRNAs were analyzed. Fig. S3 shows the efficiency of plasmid delivery. (A) Naked pSilencer-miR-320a was injected via tail every 4 weeks at a dose of 5mg/kg. The levels of miR-320a in aorta were determined at different time points by real-time PCR assays. (B) The expression of plasma miR-320a at 12 weeks determined by real-time PCR assays. Fig. S4 shows effects of miR-320a in liver. (A) Protein level expression of SRF and LXR in liver of ApoE-/- mice and wild type mice with various treatments. (B) Free fatty acid levels in liver tissue from wild type and ApoE-/- mice after plasmids injection for 12 weeks. (C) The levels of miR-320a in liver were determined at 12 weeks by real-time PCR assays. Tab. S1 shows characteristics of study cohort for miRNA microarray. 10 CAD patients, 10 subjects with CAD high risk and 10 healthy control people were included for miRNA microarray detection. Tab. S2 shows characteristics of study cohort for qRT-PCR validation. A second cohort of 20 CAD patients, 20 subjects with CAD high risk and 20 healthy control people were included for the validation of microarray assay by qRT-PCR. Tab. S3 shows characteristics of study cohort for Fig. S1. A third cohort of 50 CAD and 50 healthy control people were included for the validation of miRNAs which were already reported.

**Supporting Information Fig S1**

**
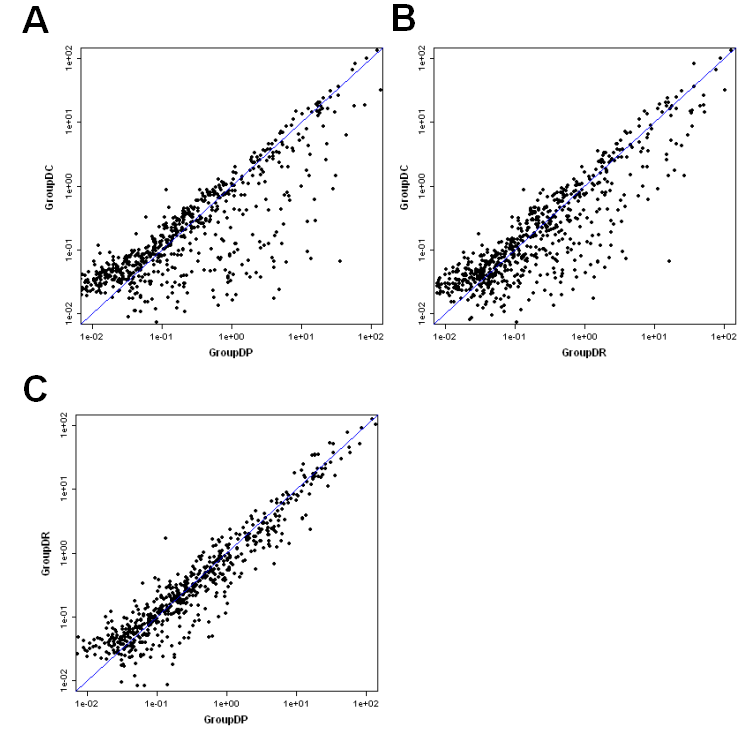
**

**Scatter plot analysis of the microarray profile of circulating miRNAs.** (A) Group DC vs. group DP; (B) Group DC vs. group DP; (C) Group DR vs. group DP.

**Supporting Information Fig S2**


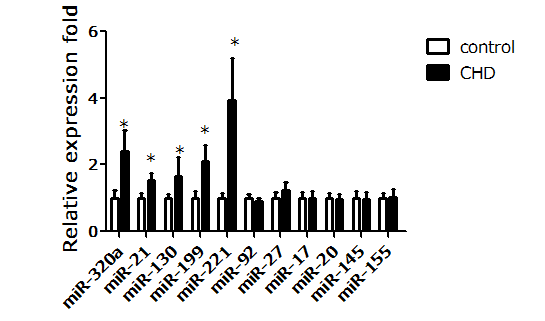


**Real-time PCR analysis of the expression of circulating miRNAs.** A third group of 50 CAD patients and 50 healthy control people were recruited, and 12 candidate miRNAs were analyzed. *p<0.05 vs. control, **p<0.01 vs. control, data are representative of 3 experiments.

**Supporting Information Fig S3**


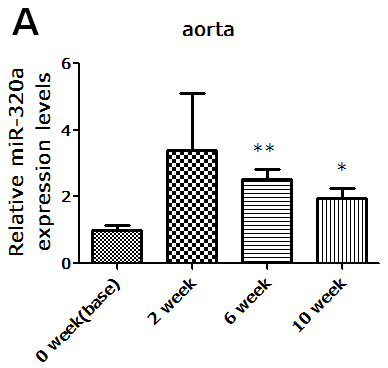
**
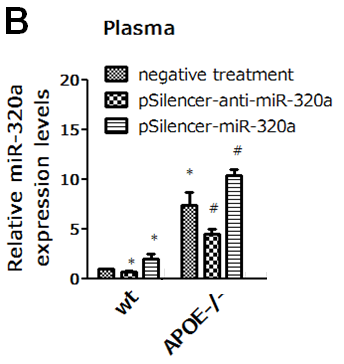
**

**The efficiency of plasmid delivery.** (A) Naked pSilencer-miR-320a was injected via tail every 4 weeks at a dose of 5mg/kg. The levels of miR-320a in aorta were determined at different time points by real-time PCR assays. *p<0.05 vs. 0 week, **p<0.01 vs. 0 week. (B) The expression of plasma miR-320a at 12 weeks determined by real-time PCR assays. *p<0.05 vs. wild type negative treatment, ^#^p<0.05 vs. ApoE^-/-^ negative treatment.

**Supporting Information Fig S4**


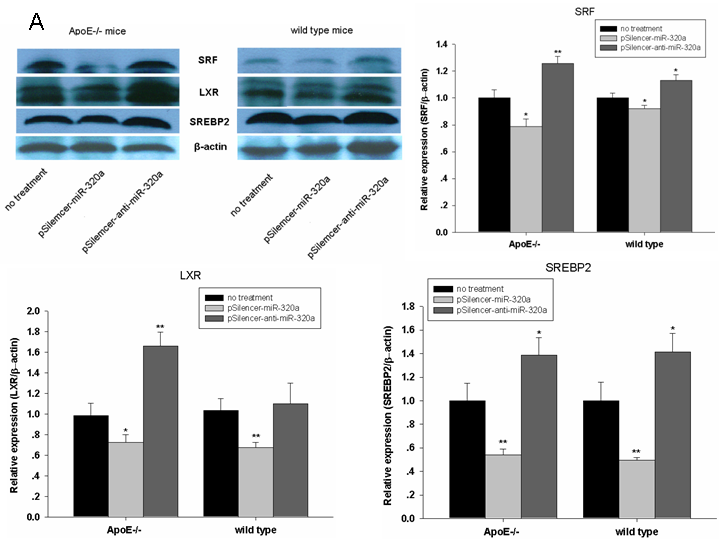


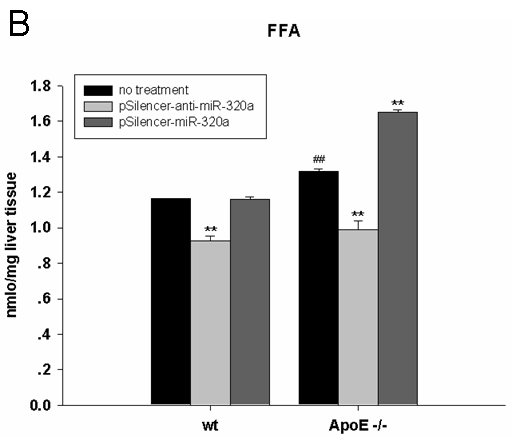

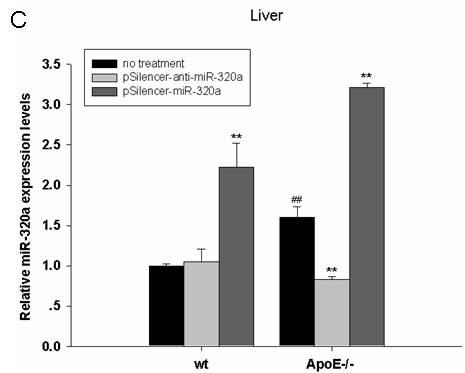


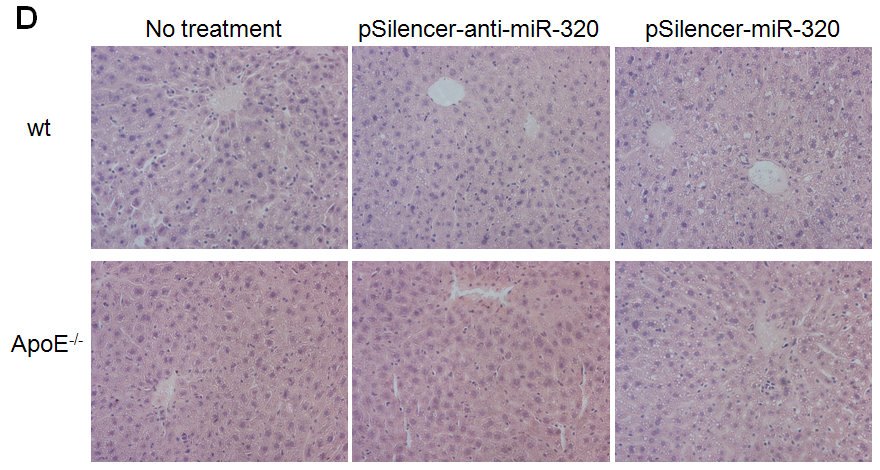


**Effects of miR-320a in liver.** (A) Protein level expression of SRF and LXR in liver of ApoE^-/-^ mice and wild type mice with various treatments, n=10. Data are expressed as relative percentage compared with control, *p<0.05 vs. same genotype with negative treatment, **p<0.01 vs. same genotype with negative treatment. (B) Free fatty acid levels in liver tissue from wild type and ApoE^-/-^ mice after plasmids injection for 12 weeks. (C) The levels of miR-320a in liver were determined at 12 weeks by real-time PCR assays. (D) HE staining of liver samples from different groups. Data are expressed as mean ± S.E. (n=10), ^##^p<0.01 vs. wild type with negative treatment, *p<0.05 vs. same genotype with negative treatment, **p<0.01 vs. same genotype with negative treatment.

**Supporting Information Table S1. Characteristics of study cohort for miRNA microarray. 10 CAD patients, 10 subjects with CAD high risk and 10 healthy control people were included for miRNA microarray detection.**

|  | **Healthy volunteers** (n=10) | **Patients with high risk** (n=10) | **Patients with CAD** (n=10) |
| --- | --- | --- | --- |
| Gender Male | 6 (60.0%) | 4 (40.0%) | 5 (50.0%) |
| Age (years) | 56.9±11.06 | 58.1±6.64 | 48.9±3.41 |
| Stable CAD for at least 4 months | 0 | 0 | 0 |
| Number of vessels  (CAD) | 0 | 0 | I: 4 (40.0%)  II: 5 (50.0%)  III: 1 (10.0%) |
| Hypertension | 0 | 8 (80.0%) | 6 (60.0%) |
| Active smoker | 2 (20.0%) | 2 (20.0%) | 5 (50.0%) |
| Adipositas (BMI > 25) | 4 (40.0%) | 3 (30.0%) | 4 (40.0%) |
| Diabetes mellitus | 0 | 1 (10.0%) | 2 (20.0%) |
| History of  AMI/PCI/ACVB/PTA/Stroke | 0 | 1 (10.0%) | 0 |
| Total cholesterol (mmol/L) | 3.95±1.09 | 4.72±0.81 | 4.35±1.67 |
| LDL cholesterol (mmol/L) | 1.92±0.76 | 2.65±2.74 | 2.55±1.42 |
| Triglycerides (mmol/L) | 2.36±2.29 | 1.60±0.82 | 1.44±0.71 |
| HDL cholesterol (mmol/L) | 1.26±0.59 | 1.34±0.30 | 1.13±0.23 |

**Supporting Information Table S2. Characteristics of study cohort for qRT-PCR validation. A second cohort of 20 CAD patients, 20 subjects with CAD high risk and 20 healthy control people were included for the validation of microarray assay by qRT-PCR.**

|  | **Healthy volunteers (n=20)** | **Patients with high risk (n=20)** | **Patients with CAD (n=20)** |
| --- | --- | --- | --- |
| Gender Male | 12 (60.0%) | 10 (50.0%) | 14 (70.0%) |
| Age (years) | 53.9±9.96 | 60.2±9.86 | 56.4±10.74 |
| Stable CAD for at least 4 months | 0 | 0 | 4 (20.0%) |
| Number of vessels  (CAD) | 0 | 0 | I: 8 (40.0%)  II: 7 (35.0%)  III: 5 (25.0%) |
| Hypertension | 0 | 18 (90.0%) | 11 (55.0%) |
| Active smoker | 6 (30.0%) | 4 (20.0%) | 11 (55.0%) |
| Adipositas (BMI > 25) | 2 (10.0%) | 4 (20.0%) | 10 (50.0%) |
| Diabetes mellitus | 0 | 4 (20.0%) | 2 (10.0%) |
| History of  AMI/PCI/ACVB/PTA/Stroke | 0 | 0 | 6 (30.0%) |
| Total cholesterol (mmol/L) | 4.09±1.03 | 4.05±0.87 | 4.29±0.98 |
| LDL cholesterol (mmol/L) | 2.16±0.75 | 2.09±0.51 | 2.38±0.76 |
| Triglycerides (mmol/L) | 1.82±1.20 | 1.78±1.38 | 1.96±1.06 |
| HDL cholesterol (mmol/L) | 1.14±0.32 | 1.21±0.34 | 1.05±0.19 |

**Supporting Information Table S3. Characteristics of study cohort for** **Supplemental Figure 1. A third cohort of 50 CAD and 50 healthy control people were included for the validation of miRNAs which were already reported.**

|  | **Healthy volunteers (n=50)** | **Patients with CAD (n=50)** |
| --- | --- | --- |
| Gender Male | 26 (52.0%) | 36 (72.0%) |
| Age (years) | 50.69±7.70 | 65.14±6.69 |
| Stable CAD for at least 4 months | 0 | 13 (26.0%) |
| Number of vessels  (CAD) | 0 | I: 15 (30.0%)  II: 21 (42.0%)  III: 14 (28.0%) |
| Hypertension | 0 | 26 (52.0%) |
| Active smoker | 12 (24.0%) | 16 (32.0%) |
| Adipositas (BMI > 25) | 10 (20.0%) | 17 (34.0%) |
| Diabetes mellitus | 0 | 12 (24.0%) |
| History of  AMI/PCI/ACVB/PTA/Stroke | 0 | 16 (32.0%) |
| Total cholesterol (mmol/L) | 4.36±1.00 | 4.30±1.19 |
| LDL cholesterol (mmol/L) | 2.72±1.30 | 2.50±1.01 |
| Triglycerides (mmol/L) | 1.67±1.49 | 1.84±1.42 |
| HDL cholesterol (mmol/L) | 1.17±0.56 | 1.03±0.30 |
